# Population structure and ancestry prediction of *Aedes aegypti* (Diptera: Culicidae) supports a single African origin of Colombian populations

**DOI:** 10.1590/0074-02760200441

**Published:** 2021-07-09

**Authors:** Yoman Monsalve, Omar Triana-Chávez, Andrés Gómez-Palacio

**Affiliations:** 1 Universidad de Antioquia, Grupo de Biología y Control de Enfermedades Infecciosas, Medellin, Colombia; 2 Universidad Pedagógica y Tecnológica de Colombia, Laboratorio de Investigación en Genética Evolutiva, Boyacá, Colombia

**Keywords:** Colombia, genetics, Aedes aegypti, population structure, microsatellites

## Abstract

**BACKGROUND:**

A previous phylogeographic study revealed two *Aedes aegypti* African-related mitochondrial lineages distributed in Colombian’s cities with different eco-epidemiologic characteristics with regard to dengue virus (DENV). It has been proposed these lineages might indicate independent invasion sources.

**OBJECTIVES:**

Assessing to Colombian population structure and to support evidence of its probable source origin.

**METHODS:**

We analysed a total of 267 individuals from cities of Bello, Riohacha and Villavicencio, which 241 were related to the West and East African mitochondrial lineages (termed here as WAL and EAL, respectively). Eight polymorphic microsatellite loci were analysed aiming population structure.

**FINDINGS:**

Results indicate substantial gene flow among distant and low-connected cities composing a panmictic population with incipient local differentiation of *Ae. aegypti* is placed in Colombia. Likewise, genetic evidence indicates no significant differences among individuals related to WAL and EAL is placed.

**MAIN CONCLUSIONS:**

Minimal genetic differentiation in low-connected *Ae. aegypti* populations of Colombia, and lack concordance between mitochondrial and nuclear genealogies suggest that Colombian *Ae. aegypti* shared a common demographic history. Under this scenario, we suggest current *Ae.* aegypti population structure reflects a single origin instead of contemporary migration, which founding populations have a single source from a mitochondrial polymorphic African ancient.

Dengue is the arthropod-borne viral disease with the fastest propagation worldwide, having a dramatic increase around 30 fold its global incidence over the past 50 years, estimated in 390 million cases by year.^1^ The primary vector of dengue virus is *Aedes aegypti*, which also transmits other viruses such as yellow fever virus, chikungunya and Zika. Similar to other vector-borne diseases, arboviruses infection must be reduced mostly by controlling vector populations.^2^ In this sense, studies about population genetic structure as well as gene flow between populations of *Ae. aegypti* are relevant to perform vector control and entomologic surveillance against these infections.^3^ For instance, if several regions in a country share a panmictic population of the same genetic background and similar/different phenotype, uniform approach for mosquito control is warranted. Contrary, if the regions happened to have different mosquito populations that are differ with respect to their genetic background and phenotypes, the uniform approaches should be tested before it would work effectively in these regions.

*Aedes aegypti* population structure has been well studied around the world, and its genetic heterogeneity and historical migration routes have been unveiled at multiple several geographic scales including global scale,^4^ regional such as in the Black-Sea region, and West Africa, and at local scale such as in California, Florida, Brazil, and Peru^5^ ^,^ ^6^ ^,^ ^7^ ^,^ ^8^ ^,^ ^9^ ^,^ ^10^ among others. Some recent examples of *Ae. aegypti* microgeographic population structure in the Americas has disclosed diverse population dynamics (i.e., migration and diversity) is placed in eclectic ecogeographical regions such as boat traffic driving migration in Peruvian Amazon,^5^ the substantial gene flow between geographically distant cities in Florida connected by an Interstate Highway,^7^ ^,^ ^8^ and the recently multiple-source invasion of diverse populations into California,^9^ ^,^ ^10^ and in several regions of Brazil.^11^ ^,^ ^12^ ^,^ ^13^

In Colombia, a first study based on random amplified polymorphic DNA-polymerase chain reaction products analysis (RAPD) reported local genetic population substructure in city of Cali, which was suggested involved in differences of vector competence and insecticide resistance ratios.^14^ Likewise, genetic analyses based on partial nucleotide sequences of mitochondrial NADH dehydrogenase 4 (ND4) gene, disclosed local microgeographic differences among individuals of municipalities of Sincelejo, Guaranda, Corozal and Sampués (within a maximum range of ~100 km) from Sucre department,^15^ ^,^ ^16^ as well as from Medellin city.^17^ These studies would suggest a complex heterogeneous population substructure is placed throughout Colombian populations mainly as consequence of local differences on selective pressures from the chemical control.

On the other hand, a more comprehensive work of *Ae. aegypti* Colombian populations reported the presence of the two mitochondrial lineages related to East and West African ancient populations, circulating in distinct frequencies across cities of Bello (BE), Riohacha (RI) and Villavicencio (VI), which having different eco-epidemiologic characteristics (i.e., dengue incidence, ecogeographical location and vector control strategies). Cities of BE, RI and VI are roughly equidistant each other (~900 km by road), and they are separated by orographic barriers such as Central and East Colombia cordilleras, and not often huge human displacement is placed among them.^18^ The suggested microgeographic structure of Colombian populations and the distinct frequency of the mitochondrial lineages observed in these cities was thought to be a consequence of possible dual or multiple invasion sources.^18^ Previous research hypothesised independent colonisation routes for *Ae. aegypti* mitochondrial lineages from different Africa sources to the New World and Latin American countries.^19^ ^,^ ^20^ ^,^ ^21^ ^,^ ^22^ ^,^ ^23^ However, most recent literature about *Ae. aegypti* populations demography, using nuclear genetic information such as polymorphic microsatellite loci and genome-wide single nucleotide polymorphisms (SNPs) approach, unveiled lack genetic concordance between differentiation patterns observed at nuclear genetic level and East, and West African mitochondrial related populations.^4^ ^,^ ^7^ ^,^ ^10^ ^,^ ^24^ ^,^ ^25^ In this sense, considering that SNPs and polymorphic microsatellite loci markers have higher genetic resolution (i.e., faster mutation rate) than mitochondrial markers, the observed mitochondrial lineage polymorphisms picture is thought occurred previous to outside Africa dispersal, and New World populations were originated by a single source from a mitochondrial polymorphic African ancient around the 16th-century, as consequence of slave trade between Africa and Americas.^4^ ^,^ ^24^

Due no comprehensive evidence for population structure of Colombian *Ae. aegypti* in this study we analysed genetic differentiation of Colombian populations, as well as addressed for whether mitochondrial West Africa lineage (WAL) and East Africa lineage (EAL) represent structured populations. Our results indicate substantial gene flow between geographically distant cities composing a panmictic population with substantial recent gene-flow of *Ae. aegypti* in Colombia, and support lack concordance between mitochondrial and nuclear genetic information. Under this scenario we propose that Colombian *Ae. aegypti* compose a unique population with incipient local genetic differentiation originated from a single source from a mitochondrial polymorphic African ancient.

## MATERIALS AND METHODS

*Sample collection and sites of the study* - *Ae. aegypti* adult mosquitoes were collected in cities of BE, RI and VI during 2012 and 2013 using entomological networks as described elsewhere.^18^ Three samplings were conducted in two neighborhoods separated by more than 1 km for each city. In order to avoid inbreeding bias in the sample less than three specimens per house were included in the genetic analyses. Entomological gathering made on private lands or in private residences were made with dweller permission and presence. A total of 267 mosquitoes, harboring 199 individuals belonging WAL and 42 to EAL were included in microsatellite analyses (Table I). Mitochondrial lineages of individuals were determined previously using partial nucleotide sequences of cytochrome oxidase I (COI) and ND4 genes and defined according to its phylogeographic clustering.^18^ The same specimens were used for mitochondrial DNA and genetic analysis performed in this work.

**TABLE I:** Geographic origin and number of *Aedes aegypti* mosquitoes analysed in this study by microsatellites, and the corresponding number of individuals related to West African lineage (WAL) and East African lineage (EAL) as previously reported^18^

Department	City (Coordinates)	Neighborhood	*n*	Individuals related to mitochondrial lineages analysed by microsatellites	
				WAL	EAL
Antioquia	Bello (6° 20‘ N 75° 35‘ W)	Cumbre	41	20	19
		Granjas	44	17	20
La Guajira	Riohacha (11° 31‘ N 72° 55‘ W)	Aeropuerto	52	45	1
		Unión	47	44	2
Meta	Villavicencio (4° 04‘ N 73° 40‘ W)	Popular	38	34	0
		Porfía	45	39	0
Total			267	199	42

*n*: number of *Aedes aegypti* mosquitoes analysed.

*DNA extraction and microsatellite genotyping* - Genomic DNA was extracted from each adult mosquito following an insect DNA extraction protocol.^26^ Microsatellite loci 1132CT1, 176TG1, 145TAAA1, 462GA1, 109CT1, 88AT1,^27^ AG5 and AC5^28^ were amplified in four multiplex polymerase chain reaction (PCR) (termed M1 - M4) according to its product size and using different dye set in forward primers [Supplementary data (Table I)]. PCR reactions for multiplex M1, M2 and M3 were conducted in a final volume of 25 µL using 3 µL of DNA template (~10 ng/µL), 1X PCR buffer (0.1 M Tris-HCl, 0.5 M KCl, and 0.015 M MgCl_2_, pH 8.3), 1.5 mM MgCl_2_, 0.2 mM dNTP, 0.2 µM of each primer, and 1 U of *Taq* DNA polymerase (Thermo Scientific, Waltham, MA, USA). For M4 multiplex, PCR reaction was conducted in a final volume of 20 µL using 1.5 µL of DNA template (~10 ng/µL), 1X PCR buffer, 1.5 mM MgCl_2_, 0.2 mM dNTP, 0.025, and 0.5 µM of forward and reverse primers respectively, and 0.75 U of *Taq* DNA polymerase (Thermo Scientific, Waltham, MA, USA). The fragments were amplified following thermal cycling conditions previously reported.^28^ Amplified fragments were visualised on 2% gel agarose stained with ethidium bromide and purified products were sent for DNA fragment detection using 400HD as internal standard size marker at Macrogen Inc., Korea. Fragment size determination with one bp resolution and allele classification were performed with Geneious v.8 software.^29^

*Genetic diversity of Ae. aegypti populations* - We used GenoDive v.3.0^30^ to test deviations from Hardy-Weinberg equilibrium (HWE) genotypic expectations and FSTAT v.2.9.4 ^31^ to test significant linkage disequilibrium (LD) between locus pairs. Local false discovery rate test (fdr) was performed for correcting statistical significance for multiple comparisons, fdr was calculated using 1,000 iterations in fdrtool package for R.^32^ We screened presence of null alleles and genotyping errors using Micro-Checker v.2.2.3,^33^ and statistical significance was assessed with 1,000 iterations.

We used GenoDive v.3.0^30^ for estimating gene diversity index such as average of number of alleles (N_a_), effective number of alleles (N_e_), observed (H_o_), and expected (H_e_) heterozygosity, and inbreeding coefficient (*G*_is_). Statistical significance was assessed with 1,000 permutations as implemented in GenoDive v.3.0.^30^

*Genetic structure of Ae. aegypti populations* - We test population genetic subdivision using the *F*_*ST*_ analogue *G*_*ST*_ ,^34^ which is calculated by relating the genetic diversity within populations to the overall genetic diversity using GenoDive v.3.0.^30^ Significance was calculated by bootstrapping with 1,000 permutations. In order to check the effect for possible null alleles in the genetic population subdivision calculated, estimates for null allele frequencies and unbiased *F*_*ST*_ calculation was restricted to visible allele sizes (so-called as ENA method) in FreeNA software.^35^ Significance was tested by *t* test using 1,000 iterations. A Mantel test was also performed in GenAlEx v.6.5 on geographic (at neighborhood) and genetic distance (pairwise *phi*PT) using 1,000 permutations.^36^

Genetic structure of the complete data set without an *a priori* assignation to a geographical origin was evaluated using the Bayesian assignment approach as implemented in STRUCTURE v.2.3.3.^37^ ^,^ ^38^ Since we assume each individual has ancestry from one or more of K genetically distinct sources, we employed the Admixture ancestry model with correlated allele frequency to find the number of genetic clusters (K). We evaluated the genetic clusters creating a batch run with K = 2 (considering WAL and EAL as possible different genetic clusters), K = 3 (assuming three cities as different genetic clusters), and K = 4, 5, and 6 (assuming between and within cities (i.e., neighborhoods) as different genetic clusters). Ten replicates per each K with 500,000 iterations per run and a burn-in of 50,000 runs were performed, and a graphical representation of the results was obtained using Distruct v.1.1.^39^ We determined the optimum K number using the ΔK criterion^40^ as implemented in Structure Harvester v.0.6.94.^41^ Individuals were considered assigned to a specific cluster if the proportion of ancestry was ≥ 80% otherwise the individual was considered unassigned.

*Relationship inference and demographic analysis of Ae. aegypti populations* - We estimate genetic relationship between individuals of Colombian populations as well as the relationship grade within Bello individuals belonging mitochondrial lineages, using the kinship (*ks)* coefficient^42^ ^,^ ^43^ in GenoDive v.3.0.^30^ Estimated *ks* values were categorised according to Iacchei et al.^44^ as follows: unrelated individuals (*ks* < 0.0475); quarter-siblings (0.0475 < *ks* < 0.09375); half-siblings (0.09375 < *ks* < 0.1875); full-siblings (0.1875 < *ks <* 0.375); and nearly identical (*ks* > 0.375). We test normality of data by Anderson-Darling test, and statistical significance (p < 0.05) of *ks* values among geographical demes (i.e., among and within cities) as well as between lineages using Kruskal-Wallis test in *dplyr* package in R.

**Fig. 1:**
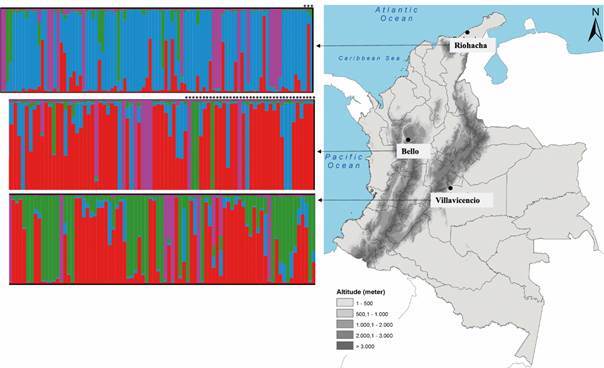
map of Colombia and genetic ancestry membership of *Aedes aegypti* to genetic clusters (K = 4) found in cities of Bello, Riohacha and Villavicencio. Each bar represents an individual and the colour is the respective ancestry component to each cluster. Asterisk (*) denotes East African lineage (EAL) related individuals as previously reported,^18^ see further details in Table I.

We used a Bayesian coalescence-based algorithm in order to address the directionality of gene flow among cities according to migration rates using Migrate-n v.4.4.4.^45^ We tested five migration models assuming similar population sizes (i.e., estimated Theta) in the three populations [Supplementary data (Fig. 1)]: (i) full model bi-directional gene flow, (ii) one-directional gene flow out of BE to RI and VI, and bi-directional gene flow between them, (iii) one-directional gene flow from RI to VI and BE, and bi-directional gene flow between them (iv) one-directional gene flow from VI to BE and RI, and bi-directional gene flow between them (v) the null model of panmixia (i.e., the samples composes a single population with constant -not-estimated- migration rate). We used standard microsatellite parameters, with uniform prior distribution set for both Theta (min: 0 to max: 1000, and delta of 10) and M (min: 0 to max: 100, and delta of 10) similar for all populations, search strategy was performed in three independent runs and parameters were: 10,000 recorded steps [a], 10 sample increment [b], and 1 concurrent chains (replicates) [c] that summarises 1,000,000 of visited parameters [a*b*c] with a burn-in of 1,000 runs using a static heating scheme of four chains with default temperature parameters. We selected the suitability of parameters used by checking the convergence of posterior probabilities distribution over all loci and effective sample size (ESS) > 200. We selected the best model test according to the log Bayes factor (LBF) based on the accurate marginal likelihoods of the Bezier approximation score generated for the five models.^46^

## RESULTS

*Microsatellite description of Ae. aegypti of Colombia* - All eight loci showed less than 15% of missing data and any out 267 individuals showed above 50% of missing data, so all individuals were included in further analyses (an overall of 89 per city; Table II). After local fdr correction p-values obtained in multiple comparisons no significant HWE nor LD deviations was observed in any population-by-locus comparison [p > 0.05; Supplementary data (Table II)]. No genotyping errors such as allele dropouts or stuttering were detected for any locus, but presence for possible null alleles having frequencies ranking between 2% to 18% were estimated in some loci across cities [Supplementary data (Table II)].

All loci were polymorphic showing a mean number of alleles (N_a_) per locus of 14.5, a mean effective number of alleles per locus (N_e_) of 3.7, mean observed heterozygosity (H_o_) of 0.53, and expected heterozygosity (H_e_) of 0.67, and overall global inbreeding index (*Gis)* of 0.21 (Table II). Most of loci showed highly variable positive *G*_*IS*_ values indicating heterozygote deficit (Table II).

**TABLE II:** Summary of variability parameters by population and by locus

Cities	*n*	N_a_	N_e_	H_o_	H_e_	*G*_*IS*_
Bello	85	9.01	3.78	0.49	0.65	0.24
Riohacha	99	11.38	3.96	0.56	0.69	0.20
Villavicencio	83	9.50	3.74	0.53	0.67	0.20
Overall	89	14.5 ± 2.2	3.67 ± 0.69	0.53 ± 0.07	0.67 ± 0.08	0.21 ± 0.04
Locus		N_a_	N_e_	H_o_	H_e_	*G*_*IS*_
1132CT1		22	5.74	0.67	0.83	0.19
462GA1		20	3.23	0.42	0.69	0.39
176TG1		18	3.26	0.69	0.69	0.02
145TAAA1		8	3.09	0.49	0.68	0.29
19CT1		4	1.18	0.07	0.16	0.52
88AT1		19	5.17	0.67	0.81	0.18
AG5		12	3.98	0.62	0.76	0.18
AC5		13	3.65	0.61	0.73	0.17
Overall		14.5	3.67	0.53	0.67	0.21

*n*: number of individuals per population; N_a_: number alleles; N_e_: effective number of alleles; H_o_: observed heterozygosity; H_e_: expected heterozygosity; *G*_*IS*_: inbreeding coefficient.

*Genetic structure analysis of Ae. aegypti in Colombia unveils low geographical differentiation* - *G*_*ST*_ statistics indicate significant, but low genetic differentiation among the three populations based on estimated overall *G*_*ST*_ (*G*_*ST*_ = 0.031; p < 0.001). Pairwise comparisons among cities indicated lower genetic differentiation between BE and VI (*G*_*ST*_ = 0.028; p < 0.001), followed for a moderate between BE and RI (*G*_*ST*_ = 0.052; p < 0.001), and RI and VI (*G*_*ST*_ = 0.056; p < 0.001). The unbiased *F*_*ST*_ per locus based on ENA correction including null alleles was not significant different from the *F*_*ST*_ estimated (*t =* 0.025; p > 0.05), indicating possible null alleles present in sample do not affect the result observed [Supplementary data (Table III)]. A Mantel test on the whole dataset showed significant but weak correlation (R^2^ = 0.02, p *<* 0.001) between genetic (*phi*PT) and geographic distance (y = 11.54x + 236.85).

**Fig. 2:**
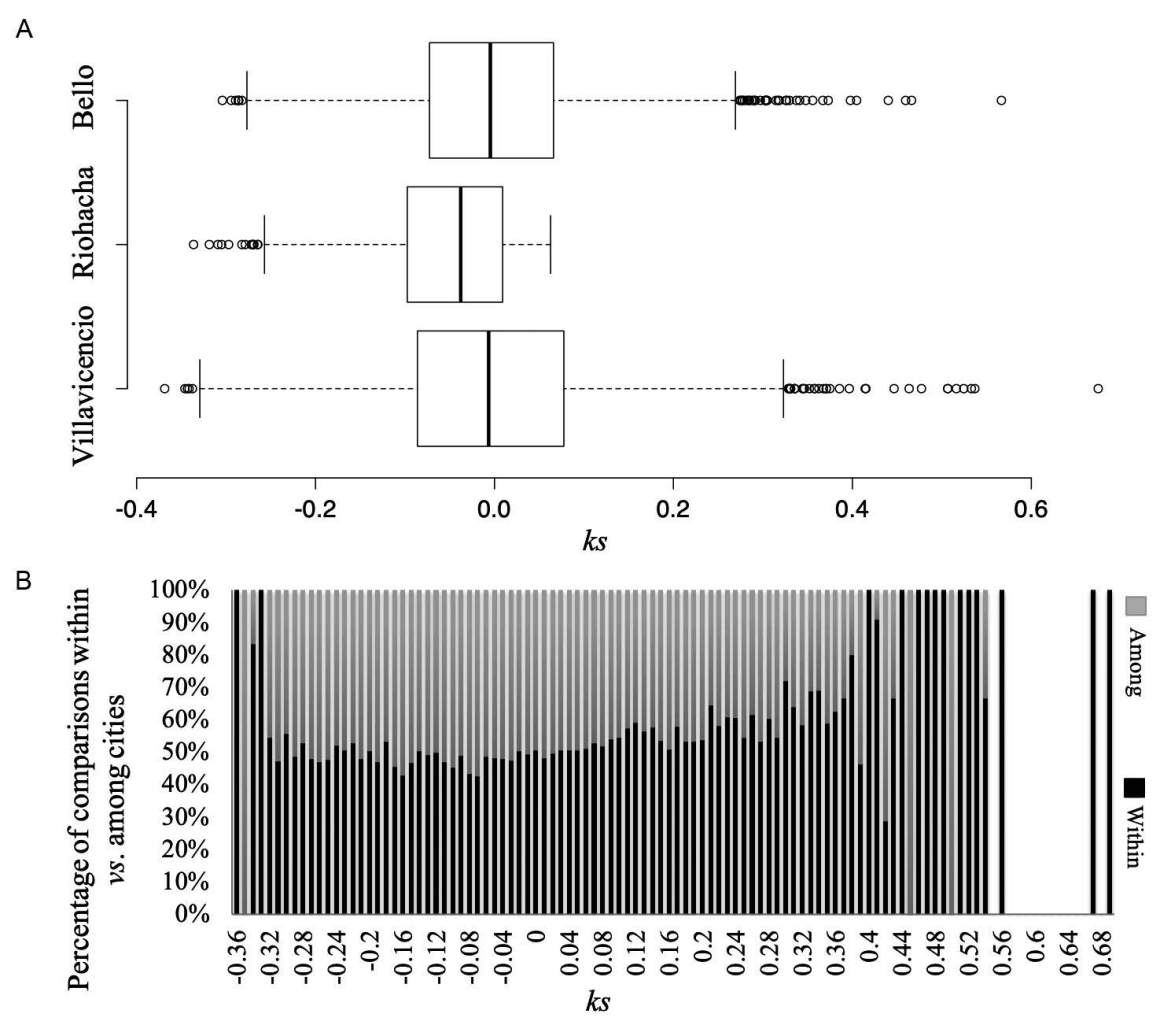
Kinship coefficient (ks) of *Aedes aegypti* individuals from cities of Bello, Riohacha and Villavicencio. (a) Boxplot of ks values estimated between individuals within cities. (b) Percentage of ks values estimated between individuals among and within cities.

Genetic structure based on Bayesian clustering indicated that the optimal clustering number is K = 4 according to the highest ΔK value obtained [Supplementary data (Fig. 2)]. However, after assignment test according to a proportion of ancestry ≥ 80%, the highest number of individuals were grouped into main clusters (Fig. 1). Out the total individuals 27.3% were not assigned to any of the four genetic clusters. Cluster 1 was composed by BE (60%), and VI (30%); cluster 2 was mostly composed by RI (57%); cluster 3 by VI (27%); and cluster 4 showed similar percentages from individuals from the three cities [Fig. 1, Supplementary data (Fig. 3)]. Otherwise, individuals did not cluster based on whether they are related to the WAL or EAL, even assuming 50% of genetic mixture per individual [Supplementary data (Table IV)].

*Demography of Ae. aegypti indicates high gene-flow among populations and no genetic differences between individuals related to WAL and EAL in Colombia* - Kinship coefficient (*ks*) for pair-wised individuals fitting to a normal distribution [Anderson-Darling normality test; A = 0.3855, p = 0.386; supplementary data (Fig. 4A)]. Median value for the entire dataset was *ks* = -0.01529, indicating most of individuals are unrelated (*ks* < 0.09375), as expected in a wide roughly panmictic population. Overall kinship coefficient for cities was not significant (Kruskal-Wallis *X* ^*2*^ = 3545.63, df = 3541, p = 0.4749; Fig. 2A), and *ks* values within-cities were comparatively higher than those calculated for individuals among-cities (Fig. 2B). No significant differences (Kruskal-Wallis *X* ^*2*^ = 1.1273, df = 1, p = 0.2883) in Kinship coefficient between individuals related to mitochondrial lineages were observed [Supplementary data (Fig. 4B-C)].

Moreover, according to the lowest LBF estimated from marginal likelihood of Bezier score (marginal likelihood = -78119.58), the best probability model (> 99.9%) test was for constant migration rate model (v), followed by similar values observed in those one-directional gene-flow based models (model (ii), marginal likelihood. = -107228.81; (iii), marginal likelihood = -108560.43, and (iv), marginal likelihood = -140668.89), and finally for the three cities for-bidirectional model (i) (marginal likelihood = -152279.34). According this result any number of migrants per generation can be accurate estimated from populations, which indicates strong evidence for possible past or recent gene-flow among the three cities.

## DISCUSSION

Here we disclose genetic population structure of Colombian *Ae. aegypti* populations from three endemic cities for relevant arboviruses such as dengue, Zika and chikungunya. Our results indicate *Ae. aegypti* Colombian population composes a panmictic population with substantial recent gene-flow among distant cities with minimal evidence of isolation-by-distance process. Furthermore, result derived from Bayesian clustering analysis where four moderated mixed genetic cluster were found as well as low values of *G*_*ST*_ index between populations, and relatively low heterozygosity observed throughout in all loci analysed would indicate high inbreeding or possible Wahlund effect is placed within populations. Likewise, high within-cities Kinship values, and null model chosen as the best scenario, indicates local demographic dynamics could driving incipient genetic differentiation processes as observed in some individuals from the three cities.

The three cities analysed BE, RI and VI are distant, separated by northern Andean cordilleras, and low human displacement is placed among them. Because, the main source for *Ae. aegypti* dispersal between distant localities is passive migration associated to human movement, results raised here suggest at least two possible not mutually exclusive hypotheses can be drawn: (i) a sustained-continuous migration between intermediate populations exists without isolation by distance evidence (i.e. patchy metapopulation structure), (ii) the population structure is retained from an ancient population invasion occurred in the recent past, it means a large founding population that rapidly spread throughout Colombia. Thus, based on our results, we conclude that microsatellite data present here are probably mirroring the ancient event of single origin instead of contemporary migration among populations. However, although this is a plausible hypothesis, further studies involving broader populations and using more resolutive markers still need to be tested.

Microgeographic structure of Colombian populations has been slightly traced so far, but no clear picture of population demographic has been supported. Several studies at local level using dominant genetic markers such as RAPDs,^14^ and nucleotide sequence analyses of mitochondrial genes, such as COI^17^ and NADH dehydrogenase subunit 4 (ND4)^15^ ^,^ ^16^ unveiled Colombian *Ae. aegypti* populations show moderated population differences at spatiotemporal scales, and it was suggested as a consequence of local pressures.

A previous research reported heterogenous distribution of mtDNA haplotypes in those cities, which have distinct dengue incidences and vector control strategies, for instance in BE city not often chemical control is applied but community-based and biological control programs are major used strategies, unlike RI and VI where insecticide-based control is often performed.^18^ In that work the authors did not support conclusive evidence for geographic or temporal genetic structuration between populations but rather the phylogeographic analysis indicated two main mitochondrial lineages related with West and East populations of Africa are differentially distributed in these Colombian cities, where in fact, both were collected in same houses in BE city.^18^ Here we support additional evidence for lack genetic concordance between local differentiation patterns observed at nuclear genetic level and West and East African mitochondrial related populations.^4^ ^,^ ^7^ ^,^ ^10^ ^,^ ^24^ ^,^ ^25^.

West and East related African mitochondrial lineages have widely been reported throughout most of South American countries such as Peru,^20^ Venezuela,^21^ Argentina,^47^ Bolivia,^23^ Brazil,^22^ ^,^ ^48^ and more recently in Ecuador.^49^ In most of the cases, authors suggested multiple invasion sources for American populations occurred, which would explain differences in frequency for mitochondrial haplotypes observed throughout South America,^23^ ^,^ ^50^ ^,^ ^51^ including Colombia.^18^ However more recent research about historical demography and global genetic structure of *Ae. aegypti* using genome-wide and highly genetic resolutive markers such as polymorphic microsatellite loci and SNPs, have indicated a single source in the native range to the New World invasive range around the 16th-century, as consequence of slave trade between Africa and Americas.^4^ ^,^ ^10^ ^,^ ^24^ ^,^ ^25^ Because mitochondrial genes show lower genetic resolution than SNPs and microsatellites, we conclude that the observed differentiation between mitochondrial lineages in Colombia does not evidence dual or multiple sources for Colombian populations, rather our observations indicate Colombian *Ae. aegypti* founding population is coherent with a single invasion source of a large mitochondrial polymorphic African related population that rapidly spread throughout Colombia. This hypothesis does not exclude scenarios for multiple introductions nor colonisation routes followed by high contemporary gene flow in Colombian populations.

Despite results agreeing with previous results suggesting lacking geographic structure of Colombian populations, it is worth notice since these studies were relied on relatively small genetic markers number (i.e., partial mitochondrial COI and ND4 genes, and eight microsatellites), therefore we suggest further studies using more resolutive genetic markers must be conducted to assessing inter-population dynamics at a finer scale of *Ae. aegypti* Colombian populations. Recent advances in next-generation sequencing technology show promising results in resolving population structure. Rašić et al.^52^ showed clearer separation of genome-wide SNP genotype profiles among *Ae. aegypti* populations that originated from different locations compared to microsatellite markers, and analyses for genetic relatedness of individuals from same populations was more accurately estimated when RAD-based genetic data was employed. Additionally, Lee et al.^10^ utilised millions of SNPs from whole genome sequencing data and showed clearer separation of populations in close proximity (20 km apart) compared to a study based on ~25K SNPs.^9^ Therefore, the use of alternative methods such as those based on high throughput data may contrast this finding and opening the possibility of pursuing more accurate results for population demography and history of Colombian populations.
