# Supplementary material for: Population structure and ancestry prediction of Aedes aegypti (Diptera: Culicidae) supports a single African origin of Colombian populations
Source: Mem Inst Oswaldo Cruz. 2021 Jul 9;116:e200441. doi: 10.1590/0074-02760200441 (PMC8279122; doi:10.1590/0074-02760200441)
Supplement: Supplementary file 1 [file 1678-8060-mioc-116-e200441-s.pdf]

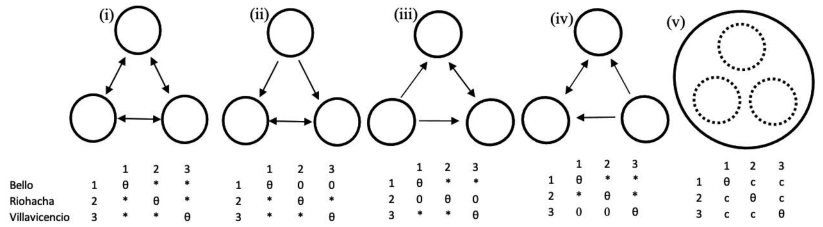

Fig. 1: population schemes for gene-flow arbitrary models of Colombian *Aedes aegypti* populations analysed and corresponding matrices as used in Migrate-n v.4.4.4.[45]

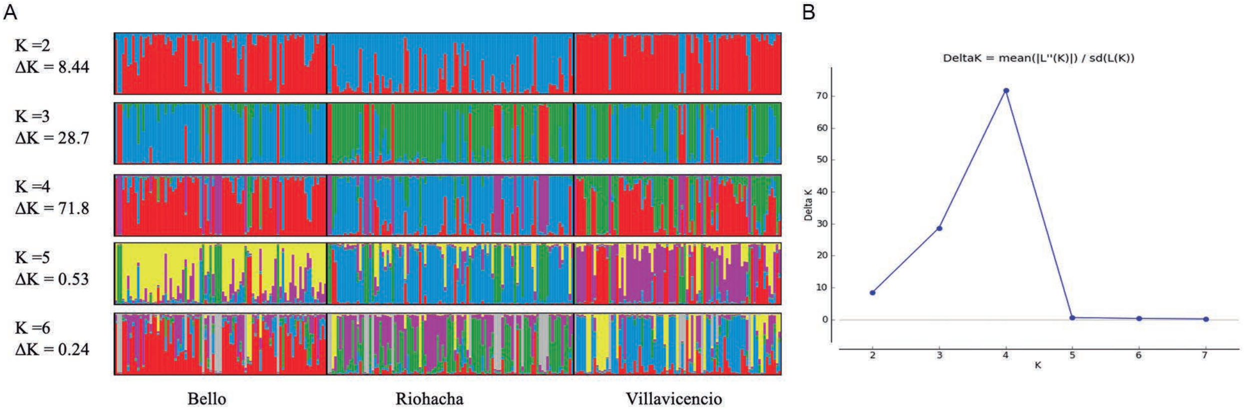

Fig. 2: Bayesian cluster analysis for Colombian populations of *Aedes aegypti*. (A) Bayesian inference of genetic clusters in *Ae. aegypti* from cities of Bello, Riohacha and Villavicencio, and harboring individuals related to WAL and EAL as previously reported.[18] Each bar represents an individual and the colour is the probability with which each individual was assigned to each cluster tested (K = 2 to K = 6, see text). B) Rate of change in the log probability of the likelihood associated to each one of the genetic clusters evaluated (ΔK).

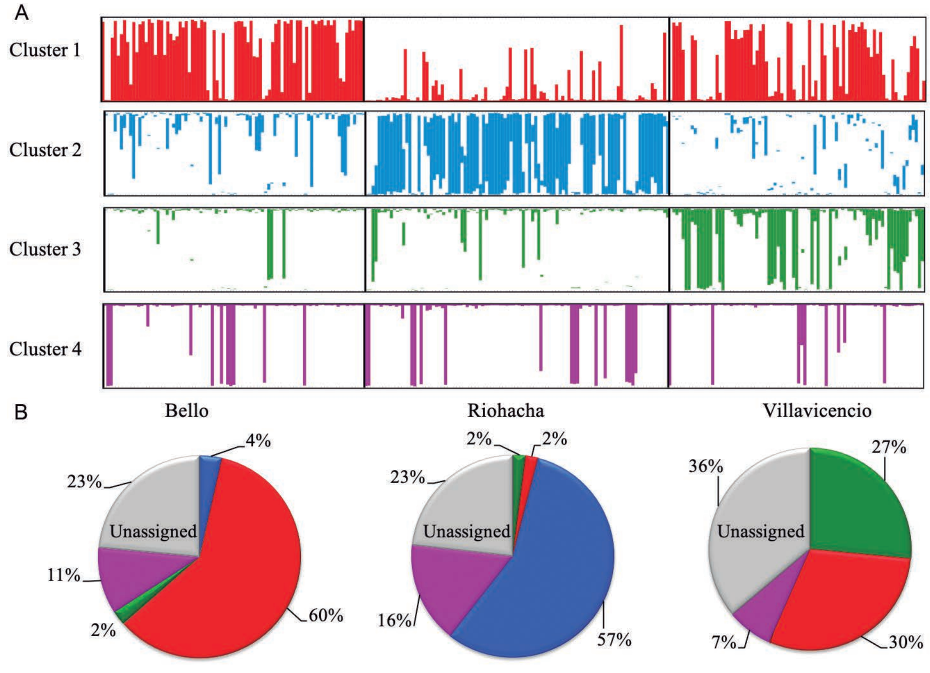

Fig. 3: Bayesian cluster analysis for Colombian populations of *Aedes aegypti*. (A) depicting assignment for individual to each genetic clusters and (B) their respective proportion in cities of Bello, Riohacha and Villavicencio.

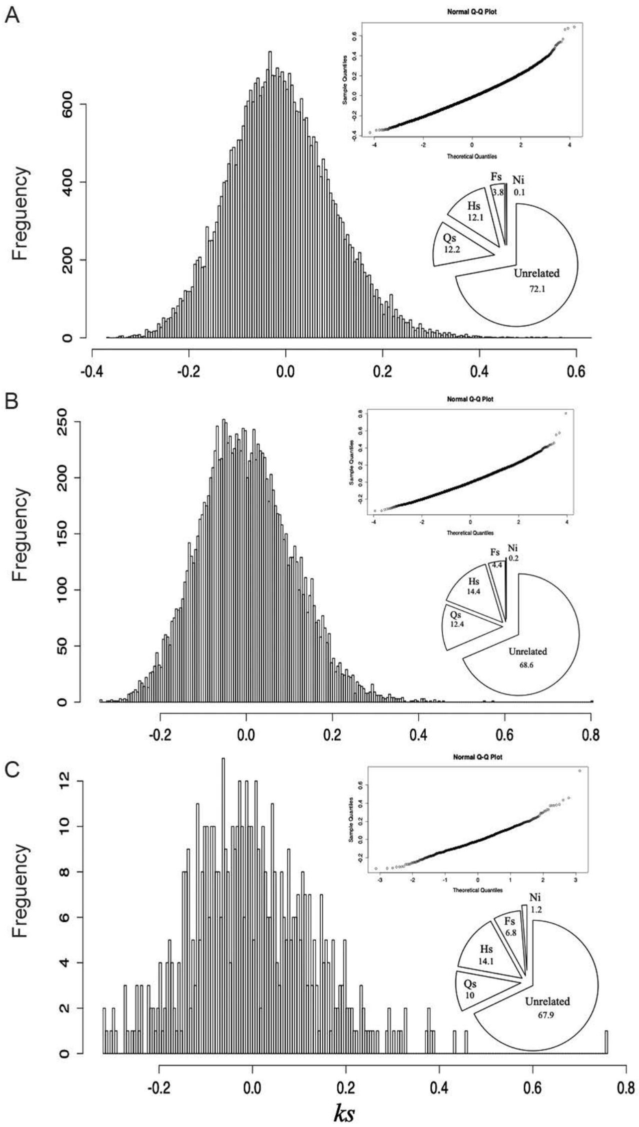

Fig. 4: histograms depicting number of kinship (*ks*) pairwise comparisons for *Aedes aegypti* related to West African lineage (WAL) and East African lineage (EAL) found in Colombia.[18] Kinship values distribution, normal Q-Q plot and percentage of kinship categories for (A) the total data set, (B) WAL and (C) EAL of *Ae. aegypti* reported in Colombia. Kinship categories and notations is as follows: Unrelated ($ks < 0.0475$); Qs = quarter-siblings ($0.0475 < ks < 0.09375$); Hs = half-siblings ($0.09375 < ks < 0.1875$); Fs = full-siblings ($0.1875 < ks < 0.375$); and Ni = nearly identical ($ks > 0.375$).

TABLE I

Multiplex polymerase chain reaction (PCR), primers and source for eight microsatellite loci used in *Aedes aegypti* from Colombia

Multiplex	Locus	Primers	Size (bp)	GenBank code	Source
M1	1132CT1	F: 5′**6-FAM**-TGACGCGTTTGACGTAGTTT-3′<br>R: 5′-TCCGCAATTACGCTAACAAA-3′	147-199	GF101930.1	(27)
	462GA1	F: 5′**TAMRA**-CAAACAACGAACTGCTCACG-3′<br>R: 5′-GAATAAGTCACACGCGTCCA-3′	316-344	GF101910.1	
M2	145TAAA1	F: 5′**6-FAM**-AGCCTTGGTATGGGGAATTT-3′<br>R: 5′-AGCATTCTTAGAGCGCTGGT-3′	132-168	GF101926.1	
	176TG1	F: 5′**HEX**-CGATCGTTGAAGGCATTTTT-3′<br>R: 5′-GAGGAACGAAACGCCAATTA-3′	322-364	GF101926.1	
M3	109CT1	F: 5′**6-FAM**-ACTGAACGCCAACCAAGC-3′<br>R: 5′-AACACCATTCTTCAGCAGAT-3′	351-357	DV362806	
	88AT1	F: 5′**HEX**-CGTCGACGTTATCTCCTTGTT-3′<br>R: 5′-CCAACGCAAGATGCAAGATA-3′	216-252	DV389063	
M4	AG5	F: 5′**6-FAM**-TGATCTTGAGAAGGCATCCA-3′<br>R: 5′CGTTATCCTTTCATCACTTGTTTG-3′	134-152	-	(28)
	AC5	F: 5′**HEX**-TGGATTGTTCTTAACAAACACGAT-3′<br>R: 5′-CGATCTCACTACGGGTTTCG-3′	125-166	-	

TABLE II

Description of population-by-locus parameters of microsatellite loci used in *Aedes aegypti* from Colombia. (a) p-value for genotypic disequilibrium based on 1000 permutations; (b) Variability description

(A)

Locus1	Locus2	Bello	Riohacha	Villavicencio	All
2CT1	2GA1	0.300	0.261	0.191	0.122
2CT1	6TG1	0.622	0.017	0.371	0.085
2CT1	TAAA	0.656	0.224	0.943	0.583
2CT1	9CT1	0.146	0.191	0.117	0.049
2CT1	8AT1	0.200	0.747	0.104	0.277
2CT1	AG5	0.025	0.858	0.220	0.232
2CT1	AC5	0.082	0.074	0.221	0.017
2GA1	6TG1	0.003	0.512	0.480	0.045
2GA1	TAAA	0.229	0.430	0.661	0.374
2GA1	9CT1	0.310	0.230	0.289	0.168
2GA1	8AT1	0.546	0.369	0.961	0.780
2GA1	AG5	0.292	0.925	0.902	0.894
2GA1	AC5	0.447	0.761	0.726	0.772
6TG1	TAAA	0.020	0.027	0.022	0.001
6TG1	9CT1	1.000	0.053	0.224	0.098
6TG1	8AT1	0.489	0.055	0.636	0.236

↳

Locus1	Locus2	Bello	Riohacha	Villavicencio	All
6TG1	AG5	0.286	0.902	0.066	0.283
6TG1	AC5	0.468	0.335	0.062	0.115
TAAA	9CT1	0.721	0.281	0.231	0.249
TAAA	8AT1	0.604	0.947	0.072	0.698
TAAA	AG5	0.070	0.644	0.329	0.205
TAAA	AC5	0.734	0.971	0.277	0.885
9CT1	8AT1	0.765	0.183	0.730	0.462
9CT1	AG5	0.045	0.782	0.686	0.557
9CT1	AC5	0.059	0.337	0.671	0.301
8AT1	AG5	0.212	0.337	0.035	0.045
8AT1	AC5	0.027	0.126	0.227	0.014
AG5	AC5	0.059	0.156	0.222	0.031

(B)

Cities	Locus	n	N_a	N_e	H_o	H_e	G_{IS}	Null allele estimates (Freq.)
Bello	1132CT1	84	13	7.2	0.751	0.861	0.134	0.06
	462GA1	85	10	2.8	0.353	0.637	0.451	0.18
	176TG1	83	10	2.6	0.554	0.608	0.095	0.04
	145TAAA1	82	6	3.7	0.501	0.733	0.323	0.13
	19CT1	85	2	1.0	0.024	0.023	-0.006	0.00
	88AT1	83	13	4.7	0.614	0.787	0.225	0.09
	AG5	76	11	4.8	0.566	0.791	0.291	0.12
	AC5	75	7	3.6	0.587	0.719	0.191	0.07
Mean over loci			9.0	3.78	0.493	0.644	0.207	0.09
Riohacha	1132CT1	99	16	4.1	0.606	0.755	0.202	0.10
	462GA1	99	18	3.9	0.525	0.745	0.300	0.14
	176TG1	98	15	5.9	0.806	0.831	0.036	0.05
	145TAAA1	98	7	2.5	0.367	0.592	0.384	0.12
	19CT1	96	4	1.4	0.146	0.271	0.465	0.13
	88AT1	94	16	6.1	0.671	0.837	0.204	0.09
	AG5	84	7	4.2	0.714	0.764	0.071	0.03
	AC5	83	11	3.7	0.651	0.727	0.111	0.06
Mean over loci			11.8	4.0	0.560	0.690	0.216	0.09
Villavicencio	1132CT1	76	17	7.3	0.658	0.863	0.244	0.11
	462GA1	75	13	3.3	0.401	0.697	0.431	0.17
	176TG1	80	10	2.8	0.701	0.641	-0.086	0.02
	145TAAA1	76	7	3.4	0.592	0.708	0.170	0.07
	19CT1	75	2	1.2	0.053	0.169	0.688	0.15
	88AT1	72	16	4.9	0.708	0.796	0.117	0.04
	AG5	76	7	3.2	0.579	0.692	0.169	0.09
	AC5	76	6	3.7	0.579	0.733	0.216	0.08
Mean over loci			9.8	3.7	0.530	0.662	0.238	0.09

n: number of individuals; N_a: number alleles; N_e: effective number of alleles; H_o: observed heterozygosity; H_e: expected heterozygosity; G_{IS}: inbreeding coefficient, and estimate null allele frequencies.

TABLE III

Calculated values of F_{ST} and unbiased F_{ST} restricted to visible allele sizes (so-called ENA method) for all loci microsatellite used in *Aedes aegypti* from Colombia

Locus	F_{ST}	F_{ST} (ENA correction)	Null allele estimates (Freq.)
2CT1	0.042	0.040	0.100
2GA1	0.018	0.013	0.167
6TG1	0.071	0.069	0.048
TAAA	0.048	0.038	0.124
9CT1	0.072	0.100	0.127
8AT1	0.013	0.012	0.085
AG5	0.039	0.036	0.091
AC5	0.087	0.085	0.084

TABLE IV

Assignment (with probability ≥ 80% and ≥ 50%) of individual genotypes of Colombian *Aedes aegypti* mosquitoes related to West Africa lineage (WAL) and East Africa lineage (EAL) to each one of the four genetic clusters defined by Bayesian analysis

Mitochondrial lineage	n	cluster 1 (%)	cluster 2 (%)	cluster 3 (%)	cluster 4 (%)	Total individuals assigned (%)
WAL	199	73 (41.5)	22 (12.5)	54 (30.7)	27 (15.3)	176 (88.4)
EAL	42	22 (68.8)	0 (0)	2 (6.3)	8 (25.0)	32 (76.2)
Total	241	95 (39.4)	22 (9.1)	56 (23.2)	35 (14.5)	208 (86.3)
		Assignation analysis assuming proportion of ancestry ≥ 50%				
Mitochondrial lineage	n	cluster 1 (%)	cluster 2 (%)	cluster 3 (%)	cluster 4 (%)	Total individuals assigned (%)
WAL	199	76 (40)	30 (15.8)	64 (33.7)	20 (10.5)	190 (95.5)
EAL	42	30 (71.4)	0 (0)	3 (7.2)	9 (21.4)	42 (100)
Total	241	106 (44.0)	30 (12.4)	67 (27.8)	29 (12.1)	232 (96.3)
